# Association between dietary selenium intake and depression in patients with or without stroke: a cross-sectional study

**DOI:** 10.3389/fnut.2025.1493603

**Published:** 2025-06-05

**Authors:** Shuang Wu, Zhimin Mei, Jin Gao, Songshan Chai

**Affiliations:** ^1^The Sixth Hospital of Wuhan, Affiliated Hospital of Jianghan University, Wuhan, China; ^2^General Hospital of the Yangtze River Shipping, Wuhan Brain Hospital, Wuhan, China; ^3^Zhongnan Hospital of Wuhan University, Wuhan, China

**Keywords:** dietary, selenium, depression, National Health and Nutrition Examination, stroke

## Abstract

**Background:**

Depression and stroke are life-threatening diseases with high incidence, research suggests an interaction between dietary selenium and depression and stroke. However, the relationship between dietary selenium and depression has not been adequately studied. Therefore, the purpose of this study was to assess the association between dietary selenium and depression among individuals with or without stroke.

**Methods:**

A cross-sectional study was performed using the 2011–2018 National Health and Nutrition Examination Survey dataset (*N* = 15,018). Logistic regression, interaction effect analysis, and restricted cubic spline analysis were used for statistical analyses.

**Results:**

The association between dietary selenium intake and prevalence depression differed between the non-stroke and stroke groups. Furthermore, when dietary selenium was converted into a categorical variable, there was evidence of an interaction between stroke status and selenium intake on decreasing the prevalence of depression (*p* = 0.007). What’s more, the dose–response association between dietary selenium intake and depression indicated various patterns between participants with and without stroke.

**Limitations:**

A cross-sectional study cannot be used to infer causal relationships.

**Conclusion:**

A non-linear relationship was observed in individuals without stroke, characterized by an apparent threshold of approximately 128.4 mcg/d. In contrast, no association was observed between dietary selenium intake and depression in participants with stroke. Further research is necessary to validate the present findings.

## 1 Introduction

Depression is a pervasive and debilitating mental health condition that affects over 300 million people worldwide. It is not only a significant cause of mental-health-related disabilities but is also projected to be the overall leading cause of disability overall by 2030 (1, 2). Studies have shown that stroke contribute to depression (3, 4). Stroke is one of the most widespread and destructive cerebrovascular diseases that results in human health problems. Research suggests that stroke is currently the second major cause of mortality and the major cause of high disability in middle-aged and elderly people (5). By 2030, the prevalence of stroke will increase from approximately 3% in Americans in 2012 to approximately 4% at a projected cost of nearly $200 billion each year (6). Several studies have reported that depression is one of the most common concomitant symptoms of stroke (7). Depressive symptoms increase the risk of death from stroke by 50%, severely reduces the quality of life of stroke survivors, delays the recovery of physical and social functioning and improvement of cognitive functioning (8, 9).

Recently, increasing evidence has suggested that the lack of a dietary inflammatory index can result in psychological disorders (10). An inadequate dietary selenium intake is closely associated with depression (11). Human diets contain selenium predominantly in the form of selenomethionine. Animals metabolize the ingested selenium into selenoprotein (12). In several studies, lower dietary selenium consumption was linked to a high incidence of depressive disorders (13, 14). Furthermore, available epidemiological studies indicate a relationship between the development of depression and dietary items such as fish (15), fruits and vegetables (16), dietary zinc and iron (17), and specific nutrients (18). Additionally, Lara et al. (19) found that in Brazilian countries, a higher total antioxidant intake was significantly associated with lower odds of depression.

Possibly, people with stroke consume more dietary antioxidants and have a lower risk of developing post-stroke depression (PSD) (20). In addition, a large cross-sectional study showed that dietary selenium was associated with stroke in a negative and non-linear correlation (21), which indicated that dietary selenium should be raised to a reasonable level to reduce the risk of stroke (22). The impact of dietary selenium on people with depression is a complex topic. Nevertheless, these studies did not pay attention to depressed patients with apoplexy. As a result, the aim of this research as an analysis of the association between dietary selenium consumption and depression as well as to reveal potential correlation between stratification of stroke and non-stroke. Therefore, using a sizable sample of the general population, we set out to compare the relationship between selenium intake and depression with or without stroke. Data from four separate NHANES cycles between 2011 and 2018 were used to strengthen the study.

However, the association between dietary selenium intake and depression in individuals with and without stroke remains unclear. There is a lack of comprehensive research examining these associations in a single study. It would be valuable to clarify and understand these connections to provide patients with guidance on reducing the prevalence of depression. To fill this gap, based on the National Health and Nutrition Examination Survey (NHANES), we investigated the association between dietary selenium intake and depression in both stroke and non-stroke participants in the general population. Additionally, we conducted an analysis exploring the dose–response relationship between dietary selenium intake and the odds of depression in adults with and without stroke.

## 2 Materials and methods

### 2.1 Data sources and study sample

**Figure 1:**
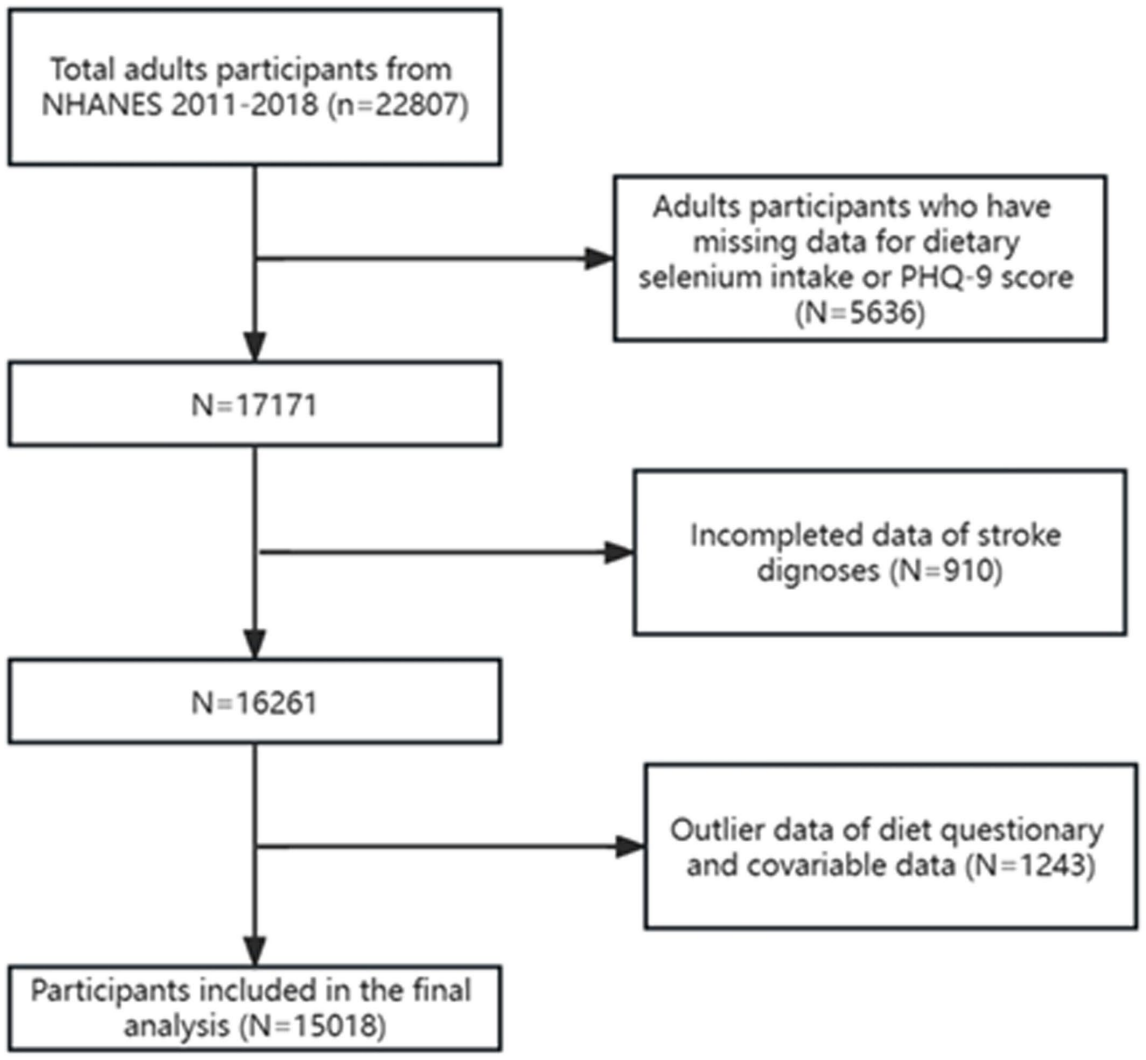
Flow chart of sample selection from the NHANES 2011–2018.

The NHANES is a collection of medical investigations intended to assess the nutritional and physical health of non-institutionalized Americans. All data for this study were publicly available on the NHANES official website (23), and NHANES data from 2011 to 2018 were used. A multistage stratified probability procedure was used to select survey participants as an accurate representation of the population (24). Comprehensive family interviews were conducted to obtain basic demographic and health history data. Physical examinations and blood sample collection were performed at a mobile examination center (MEC). Serum samples were examined at the National Center for Environmental Health’s Division of Laboratory Sciences of the Centers for Disease Control and Prevention. The National Center for Health Statistics Research Ethics Review Board approved this study. The participants in our study participated in an interview and assessment at the MEC, and were at least 20 years old. Participants were excluded if their depression status, covariates, or stroke status were lacking. A flowchart showing the data filtration process for the study participants is shown in Figure 1.

Following NHANES analytic guidelines, we incorporated examination center sample weights and accounted for the complex survey design.

### 2.2 Depressive symptoms

Participants in the NHANES database completed the Patient Health Questionnaire (PHQ-9), which was used to assess their depression status and its severity. The PHQ-9 is a self-assessment depression screening tool based on the Diagnostic and Statistical Manual of Mental Disorders, Fourth Edition, which contains nine items reflective of major depressive disorders. It is a nine-item screening tool that assesses how frequently diverse depressive symptoms have occurred over the past two weeks (25). The overall score, which varies from 0 to 27, can be divided into a variety of cut-off points, with 0 meaning “not at all,” 1 meaning “a few days,” 2 meaning “more than half the time,” and 3 meaning “almost every day.” A score of 10 was determined to be 88% sensitive and specific for major depression, suggesting moderate to severe depressive symptoms; this score was utilized as the cut-off point for participation in the depression group (26).

### 2.3 Dietary selenium intake

Dietary intake data were obtained from the National Health and Nutrition Examination Survey (NHANES) database, utilizing 24-h dietary recall interviews conducted during the Mobile Examination Center (MEC) visits as part of the “What We Eat in America” program. Trained interviewers employed the NHANES computer-assisted dietary interview system to meticulously record detailed descriptions and quantities of all foods and beverages consumed by participants within the 24-h period preceding the interview. Nutrient composition, including dietary selenium levels, was calculated using standardized food composition references from the USDA Food and Nutrient Database for Dietary Studies (FNDDS) and the University of Texas Food Intake Analysis System (FIAS). Notably, nutrient estimates excluded contributions from prescription medications or dietary supplements to isolate dietary sources of selenium. The NHANES Dietary Interviewers’ Procedure Manual provides comprehensive explanations of dietary data collection (27). Participants were interviewed twice to gather information on their dietary selenium and supplement intake. The first interview was conducted in person during the mobile examination, whereas the second interview was conducted over the phone within 3–10 days. By combining the information obtained from both interviews, we calculated the total selenium intake for each participant. The average selenium intake from the two interviews was considered the participants’ overall selenium intake.

### 2.4 Identification of stroke

Stroke status was identified using the Medical Condition Questionnaire. When subjects responded “yes” to the question, “Has a doctor or other health professional ever told you that you had a stroke?” they were considered to have a stroke (28).

### 2.5 Other covariates

Based on clinical experience, univariate analysis, and a previously related publication (29), we included the following covariates as risk factors for depression: demographic variables, including age, gender, and race, were self-reported in the interview. Education level, health insurance, and the ratio of household income to poverty (PIR) were indicators of socioeconomic status. There were three categories of education level: “high school,” “high school graduate,” and “college graduate or above.” The variables relating to health included Body Mass Index (BMI) (under/normal weight: <25 kg m^−2^; overweight: ≥25 to <30 kg m^−2^; obesity: ≥30 kg m^−2^), smoking (15) (never smokes; smokers: used to smoke > 100 cigarettes but no longer did so and smoking at the time of the interview and had previously smoked >100 cigarettes), alcohol use (30) (non-drinkers; drinkers: consumed ≥ 12 drinks annually throughout their lives), physical activity and energy intake (tertiles). This study used self-reported history interview data for subjects who were at least 18 years old to determine the BMI (kg m^−2^) by measuring the participants’ height and weight and their alcohol and cigarette consumption. Furthermore, dietary energy intake was obtained from the Nutrient Database for Dietary Studies, where nutrient intake was estimated using dietary intake data from participants’ 24-h dietary recall interviews (31). Hypertension was, defined as a mean systolic blood pressure ≥ 130 mmHg, or a mean diastolic blood pressure ≥ 80 mmHg or a combination of the physician’s history of diagnosis in the questionnaire (32). Diabetes was defined as fasting blood glucose level ≥ 7.0 mmol/L, or 2-h blood glucose level ≥ 11.1 mmol/L, or glycated hemoglobin level ≥ 6.5%, or having hypoglycemic drugs or injecting insulin, or self-reported diagnosis of diabetes (32). Hyperlipidemia was defined as elevated levels of serum triglycerides (TG ≥ 150 mg/dL), total cholesterol (TC ≥ 200 mg/dL), and low-density lipoprotein cholesterol (LDL-C ≥ 130 mg/dL), along with low high-density lipoprotein cholesterol (HDL-C ≤ 40 mg/dL in males and ≤50 mg/dL in females). Another criterion for defining hyperlipidemia was the use of medications specifically designed to treat this condition. To assess the lipid profiles of the participants in our study, we measured the concentrations of TG, TC, HDL-C, and LDL-C in the blood samples of a subset of individuals who volunteered for testing at the MEC.

## 3 Statistical analysis

Descriptive analysis was calculated using survey-weighted methods, with continuous variables expressed as weighted means ± standard errors (SEs) and categorical variables presented as weighted proportions (%) with corresponding SEs. Normality distribution was assessed through Shapiro–Wilk testing (*α* = 0.05), followed by appropriate parametric analysis: independent t-tests for normally distributed variables and Kruskal-Wallis tests for nonparametric comparisons. Weighted univariate and multivariate logistic regression analyses were performed to investigate the relationship between dietary selenium intake and depression. In weighted multivariate logistic regression, dietary selenium was analyzed as a continuous and categorical variable, with model 1 adjusted for age, gender, race, education, BMI, and marital status, and model 2 adjusted for variables in model 1 plus smoking status, alcohol consumption, diabetes, hypertension, hyperlipidemia, sleeping disorders, and energy intake. Stratified analyses compared selenium-depression relationships between stroke and non-stroke subgroups, with interaction effects evaluated through likelihood ratio tests incorporating cross-product terms. A restricted cubic spline model with four prespecified knots (5th, 35th, 65th, and 95th percentiles) quantified potential non-linear dose–response relationships. Nonlinearity was formally tested using hierarchical likelihood ratio tests comparing linear and spline models. Sensitivity analyses were performed by excluding extreme selenium intake values beyond ±2SD (13.63–192.92 mcg/d) and ±3SD (2.35–237.74 mcg/d) thresholds. Multiple imputation with chained equations (MICE, 5 iterations) addressed missing covariate data (<8% missingness across variables), incorporating predictive mean matching and logistic regression imputation models.

All the analyses were performed using the statistical software packages R (http://www.R-project.org, The R Foundation) and Free Statistics software versions 1.8.1. *p*-value was considered statistically significant at <0.05.

## 4 Results

### 4.1 Baseline characteristics of the study sample

Figure 1 illustrates the participant selection flowchart. After removing those with missing data (*n* = 7,789), we selected 15,018 (weighted *n* = 98,162,065) possible participants from the NHANES for our analysis. As detailed in Table 1, stroke survivors demonstrated significantly lower median dietary selenium intake compared to non-stroke participants (89.7 mcg/d [IQR:46.88–132.52] vs. 103.65 mcg/d [IQR:58.84–148.46]). Compared with non-stroke participants, stroke survivors exhibited significantly lower daily dietary intakes of energy, calcium, protein, magnesium, phosphorus, iron, zinc, and copper, as well as reduced poverty-income ratios (PIR). Additionally, the stroke group was older and had higher body mass index (BMI) values, lower family income, and lower educational attainment. They were also more likely to be male, former smokers, non-drinkers, and physically inactivity, with higher prevalence rates of hypertension, diabetes, thyroid disorders, sleep disturbances, and cardiovascular disease (*p* < 0.05 for all comparisons). Univariate analysis (Supplementary Table S1) revealed that female sex (58.2% vs. 40.6%; OR: 1.578 [0.976, 2.552]) and obesity (OR: 1.604 [0.903, 2.848]) were positively associated with depression risk, along with smoking (OR: 2.389 [1.478, 3.861]), alcohol consumption (OR: 1.391 [0.595, 3.256]), hypertension (OR: 1.512 [0.947, 2.413]), hyperlipidemia (OR: 1.331 [0.787, 2.249]), and diabetes (OR: 1.519 [0.784, 2.941]). Conversely, higher educational attainment (OR: 0.422 [0.226, 0.787]) and elevated energy intake (OR: 0.649 [0.367, 1.149]) showed inverse associations with depression. Physical inactivity further correlated with paradoxical inverse association (OR: 0.719 [0.409, 1.263]).

**Table 1:** Characteristics of the study participants (US adults), NHANES 2011–2018.

Characteristics	Quartile 1 (2.35 ~ 76.112)	Quartile 2 (76.112 ~ 103.275)	Quartile 3 (103.275 ~ 135.9)	Quartile 4 (135.9 ~ 250)	*P*-value
No.	3,755	3,754	3,753	3,756	
Selenium intake (mcg d^−1^)	60.6 (48.7, 68.9)	89.8 (83.0, 96.4)	118.2 (110.4, 126.6)	163.2 (147.8, 187.3)	< 0.001
Age (year)	52.7 ± 18.0	50.9 ± 17.9	49.0 ± 17.2	46.0 ± 16.2	< 0.001
Age group, *n* (%)					< 0.001
20 ~ 40	1,014 (27)	1,177 (31.4)	1,270 (33.8)	1,473 (39.2)	
40 ~ 60	1,195 (31.8)	1,184 (31.5)	1,273 (33.9)	1,367 (36.4)	
≥60	1,546 (41.2)	1,393 (37.1)	1,210 (32.2)	916 (24.4)	
Sex, *n* (%)					< 0.001
Male	1,039 (27.7)	1,490 (39.7)	1954 (52.1)	2,612 (69.5)	
Female	2,716 (72.3)	2,264 (60.3)	1799 (47.9)	1,144 (30.5)	
Race, *n* (%)					< 0.001
Mexican American	435 (11.6)	460 (12.3)	470 (12.5)	561 (14.9)	
Other Hispanic	403 (10.7)	362 (9.6)	384 (10.2)	353 (9.4)	
Non-Hispanic White	1,477 (39.3)	1,547 (41.2)	1,538 (41)	1,410 (37.5)	
Non-Hispanic Black	1,014 (27)	848 (22.6)	807 (21.5)	797 (21.2)	
Other races	426 (11.3)	537 (14.3)	554 (14.8)	635 (16.9)	
Education, *n* (%)					< 0.001
Less than high school	893 (23.8)	722 (19.2)	624 (16.6)	611 (16.3)	
High school	887 (23.6)	845 (22.5)	794 (21.2)	803 (21.4)	
More than high school	1975 (52.6)	2,187 (58.3)	2,335 (62.2)	2,342 (62.4)	
Marital status, *n* (%)					< 0.001
Married/Living with partner	1,015 (27)	881 (23.5)	730 (19.5)	659 (17.5)	
Divorced/separated/widowed	501 (13.3)	483 (12.9)	549 (14.6)	563 (15)	
Never married	2,239 (59.6)	2,390 (63.7)	2,474 (65.9)	2,534 (67.5)	
BMI, Median (IQR)	28.3 (24.6, 33.4)	28.3 (24.5, 33.3)	28.7 (24.8, 33.4)	28.2 (24.4, 32.8)	0.018
BMI category, *n* (%)					0.135
<25	1,021 (27.2)	1,049 (27.9)	978 (26.1)	1,061 (28.2)	
25 ~ 30	1,191 (31.7)	1,193 (31.8)	1,192 (31.8)	1,230 (32.7)	
≥30	1,543 (41.1)	1,512 (40.3)	1,583 (42.2)	1,465 (39)	
Total cholesterol (mg dL^−1^)	189.0 (163.0, 218.0)	187.0 (161.0, 214.0)	189.0 (164.0, 215.0)	186.0 (162.0, 214.0)	0.028
Total triglyceride (mg dL^−1^)	97.0 (66.0, 138.0)	94.0 (65.0, 140.0)	97.0 (67.0, 146.5)	98.0 (66.0, 149.0)	0.123
HDL-C (mg dL^−1^)	53.0 (43.0, 64.0)	51.0 (42.0, 63.0)	51.0 (42.0, 62.0)	49.0 (41.0, 59.0)	< 0.001
LDL-C (mg dL^−1^)	110.0 (86.0, 135.5)	109.0 (87.8, 134.0)	109.0 (87.0, 134.8)	110.0 (88.0, 133.0)	0.885
Physical activity, *n* (%)					< 0.001
High (≥600)	769 (20.5)	740 (19.7)	716 (19.1)	628 (16.7)	
Low (<600)	2,986 (79.5)	3,014 (80.3)	3,037 (80.9)	3,128 (83.3)	
Energy intake (kcal d^−1^)	1289.0 (1018.5, 1600.5)	1731.0 (1460.1, 2036.9)	2080.5 (1756.0, 2452.5)	2639.8 (2214.4, 3149.1)	< 0.001
Energy intake, *n* (%)					< 0.001
Tertile 1	2,840 (75.6)	1,456 (38.8)	565 (15.1)	145 (3.9)	
Tertile 2	781 (20.8)	1732 (46.1)	1,694 (45.1)	796 (21.2)	
Tertile 3	134 (3.6)	566 (15.1)	1,494 (39.8)	2,815 (74.9)	
Smoking status, *n* (%)					< 0.001
No	2,247 (59.8)	2,196 (58.5)	2097 (55.9)	2091 (55.7)	
Yes	1,508 (40.2)	1,558 (41.5)	1,656 (44.1)	1,665 (44.3)	
Alcohol consumption, *n* (%)					< 0.001
No	742 (19.8)	605 (16.1)	456 (12.2)	371 (9.9)	
Yes	3,013 (80.2)	3,149 (83.9)	3,297 (87.8)	3,385 (90.1)	
Hypertension, *n* (%)					< 0.001
No	1954 (52)	2064 (55)	2,131 (56.8)	2,315 (61.6)	
Yes	1801 (48)	1,690 (45)	1,622 (43.2)	1,441 (38.4)	
Diabetes, *n* (%)					< 0.001
No	3,113 (82.9)	3,144 (83.8)	3,215 (85.7)	3,285 (87.5)	
Yes	642 (17.1)	610 (16.2)	538 (14.3)	471 (12.5)	
Hyperlipidemia, *n* (%)					< 0.001
No	1,076 (28.7)	1,127 (30)	1,200 (32)	1,317 (35.1)	
Yes	2,679 (71.3)	2,627 (70)	2,553 (68)	2,439 (64.9)	
Sleeping disorders, *n* (%)					0.133
No	3,253 (86.6)	3,304 (88)	3,257 (86.8)	3,241 (86.3)	
Yes	502 (13.4)	450 (12)	496 (13.2)	515 (13.7)	

BMI, Body mass index.

### 4.2 Dietary selenium intake affects the prevalence of depression among individuals with and without stroke

A significant inverse association was observed between dietary selenium intake and depression prevalence among non-stroke participants (OR [95% CI], 0.992 [0.986–0.998]), whereas no association emerged in stroke survivors (OR [95% CI], 1.006, [0.985–1.027]). After adjusted for age, gender, race, education, BMI, and marital status, the results were comparable (OR [95% CI], 0.993, [0.987–1.01]). The negative association remained significant (all *p*-value < 0.05) even after controlling for additional potential confounders such as smoking status, alcohol consumption, diabetes, hypertension, hyperlipidemia, sleeping disorders and energy intake. When analyzed categorically, participants in the highest selenium intake quartile (Q4:136.3–250 mcg/d) exhibited a 58.1% reduction in depression risk compared to Q1 (<76.75 mcg/d) (prevalence of depression: OR [95% CI], 0.419 [0.215, 0.819]) in the unadjusted model. After adjusted for age, gender, race, education, BMI, marital status, smoking status, alcohol consumption, diabetes, hypertension, hyperlipidemia, sleep disorders, and energy intake, the results were similar (OR [95% CI], 0.383 [0.173, 0.851]) (Table 2). When dietary selenium consumption was converted to a categorical variable, there was an interaction between dietary selenium and depression in both stroke participants and those without stroke (For the likelihood ratio test for the interaction *p* = 0.007) (Table 3). In Supplementary Table S2, the associations between dietary selenium intake and depression were consistent among crude data in Model 1 and Model 2, and findings from the linear regression model are consistent with those from the logistic regression. In Supplementary Table S5, the associations between dietary selenium intake and depression were consistent after adjusting for serum selenium levels (OR = 0.99, 95% CI: 0.982–0.999, *p* = 0.0278). The effect estimates for key covariates (e.g., age, smoking status, comorbidities) were unchanged, indicating robustness to the inclusion of serum selenium.

**Table 2:** Weighted odds ratios (95% confidence intervals) of depression and dietary selenium intake levels in different models among US adults, NHANES 2011–2018.

	With stroke (*n* = 545)			Without stroke (*n* = 14,473)		
	Crude	Model 1	Model 2	Crude	Model 1	Model2
Selenium intake (per-SD change)	1.006 (0.985 ~ 1.027)	1.005 (0.981 ~ 1.029)	0.997 (0.959 ~ 1.035)	0.992 (0.986 ~ 0.998)***	0.993 (0.987 ~ 1.01)****	0.99 (0.982 ~ 0.999)****
Subgroups						
Quartile 1	Reference	Reference	Reference	Reference	Reference	Reference
Quartile 2	1.618 (0.132 ~ 19.888)	1.564 (0.109 ~ 22.469)	1.498 (0.064 ~ 35.09)	0.654 (0.358 ~ 1.195)	0.689 (0.372 ~ 1.277)	0.674 (0.347 ~ 1.31)
Quartile 3	1.372 (0.086 ~ 21.944)	1.33 (0.066 ~ 26.76)	1.264 (0.036 ~ 44.963)	0.419 (0.215 ~ 0.819)****	0.462 (0.231 ~ 0.924)	0.383 (0.173 ~ 0.851)****
Quartile 4	2.044 (0.137 ~ 30.537)	1.948 (0.096 ~ 39.412)	0.816 (0.009 ~ 75.809)	0.435 (0.225 ~ 0.84)****	0.496 (0.243 ~ 1.012)	0.377 (0.147 ~ 0.968)****
P-trend	0.642	0.699	0.935	0.005	0.024	0.019

Data presented are ORs and 95% Cls. Crude model: Unadjusted crude model. Model 1: Adjusted for age, gender, race, education, BMI, and marital status. Model 2: Adjusted for variables in model 1 plus smoking status, alcohol consumption, diabetes, hypertension, hyperlipidemia, sleeping disorders and energy intake. Weighted by: 24-h dietary recall. *p < 0.0001, **p < 0.001, ***p < 0.01, ****p < 0.05.

**Table 3:** Subgroup analyses between dietary selenium intake and risk of depression in patients with or without stroke among US adults, NHANES 2011–2018.

Variable	With stroke (*n* = 545)		Without stroke (*n* = 14,473)		P for interaction
	OR 95% CI	*P*-value	OR 95% CI	*P*-value	
Dietary selenium intake (mcg/d)	1.002 (0.997 ~ 1.006)	0.508	0.994 (0.993 ~ 0.996)	<0.001	0.032
Subgroups					0.007
Quartile 1	Ref.		Ref.		
Quartile 2	1.762 (0.939 ~ 3.304)	0.078	0.689 (0.581 ~ 0.818)	<0.001	
Quartile 3	1.454 (0.686 ~ 3.082)	0.449	0.512 (0.419 ~ 0.623)	<0.001	
Quartile 4	1.198 (0.471 ~ 3.052)	0.704	0.461 (0.362 ~ 0.587)	<0.001	
Trend test		0.636		<0.001	

Data presented are ORs and 95% CIs. Adjusted for variables in model 1 plus smoking status, alcohol consumption, diabetes, hypertension, hyperlipidemia, sleeping disorders and energy intake.

### 4.3 Sensitive analysis and threshold effect analysis

**Figure 2:**
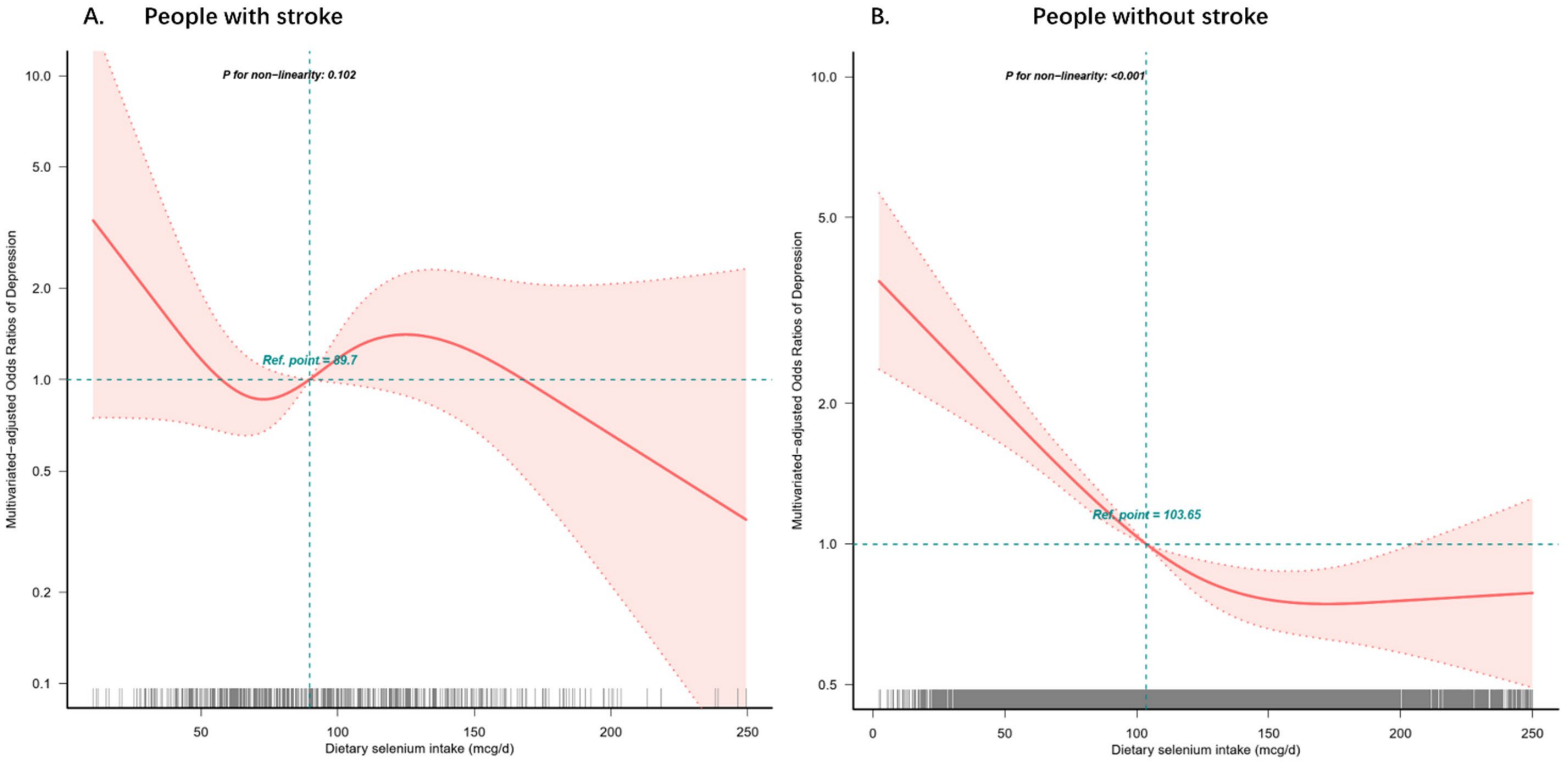
Restricted cubic spline model of the odds ratios of dietary selenium intakes with depression with stroke **(A)** or without stroke **(B)**. Adjusted for age, gender, race, education, BMI, and marital status, smoking status, alcohol consumption, diabetes, hypertension, hyperlipidemia, sleeping disorders and total daily energy intake. The dashed lines represent the 95% confidence intervals.

Restricted cubic spline analysis regression with four knots (5th, 35th, 65th, 95th percentiles) revealed significant non-linear associations between dietary selenium intake and depression risk, stratified by stroke history (Figure 2). Among individuals without stroke, a J-shaped relationship was observed across the selenium intake spectrum (2.35–250 mcg/d), with minimal depression risk at 128.4 mcg/d (nadir point) (all *p* < 0.001, P nonlinearity = 0.001, Figure 2A), but not in the stroke group (Figure 2B).

In the sensitivity analyses, the results remained stable after excluding participants with dietary selenium intakes > 2.35 mcg/d (mean ± 3SD) (Supplementary Table S3). The prevalence of depression decreased significantly with an increase dietary selenium intake in the non-stroke group (OR [95% CI], 0.993 [0.991, 0.995], *p* < 0.001), whereas the relationship was not significant in the stroke group (OR [95% CI], 0.997 [0.988, 1.005], *p* = 0.459). When dietary selenium was transformed into a categorical variable, significant effect modification of dietary selenium intake on the prevalence of depression with or without stroke (for the likelihood ratio test *p* = 0.008). When subjects with dietary selenium intakes > 13.63 mcg/d (mean ± 2SD) were excluded (Supplementary Table S4), the prevalence of depression was substantially lower in the non-stroke group as dietary selenium increased (OR [95% CI], 0.991 [0.989, 0.994], *p* < 0.001). However, the decrease in the stroke group was not significant (OR [95% CI], 0.999 [0.99, 1.008], *p* = 0.815). Similarly, the interaction likelihood ratio test showed a *p* value of 0.002 when dietary selenium was converted into a categorical variable.

## 5 Discussion

The cross-sectional study reveals a critical distinction in the relationship between dietary selenium intake and depression based on stroke history. The principal findings demonstrate a significant non-linear inverse association between selenium consumption and depression prevalence in non-stroke individuals (*p* < 0.05), with those in the highest selenium intake quartile (128.4 mcg/d) showing 53.9% lower depression risk compared to the lowest quartile (OR: 0.461, 95% CI: 0.362–0.587). Notably, this protective association was absent in stroke survivors, suggesting potential pathophysiological modifications of selenium’s neuroprotective effects following cerebrovascular events.

The association between dietary selenium intake and depression has been studied extensively. Zhang et al. (33) found null effects for blood selenium, while Wang et al. (34) reported benefits for dietary selenium. However, epidemiological evidence in the general population is inconsistent, with some studies suggesting a negative or nonexistent association (35). While our observation of selenium’s antidepressant potential in the general population aligns with US cohort studies (13, 36) and Australian case–control data (14), the null association in post-stroke depression (PSD) contrasts with Xu’s U-shaped correlation in Chinese stroke patients (20). Similarly, several studies have revealed an association between dietary selenium intake and a decreased likelihood of stroke (37, 38), however, little attention has been paid to the association between dietary selenium intake and depression with or without stroke. This discrepancy may reflect population-specific selenium status variations or differential stroke pathophysiology across ethnic groups. Importantly, our stratified analysis addresses a critical knowledge gap—no prior investigation has concurrently examined this relationship in both stroke and non-stroke populations using nationally representative data.

Wang et al. (39) focused on depressed adults, whereas our study stratified by stroke history. We replicated their antioxidant synergy findings in non-stroke adults but observed null effects in post-stroke populations. Several studies have indicated that selenium supplementation may improve the antioxidant capacity in individuals with coronary artery disease (40). However, the potential of increasing selenium intake to prevent stroke and related complications remains unclear. To date, few retrospective cohort studies have examined the association between dietary selenium intake and stroke incidence. Unfortunately, these studies provide inconclusive results. Previous studies have investigated the effects of selenium treatment on cellular redox balance, lipid peroxidation, and neuronal protection during ischemia (41, 42). However, there have been conflicting findings from observational and interventional studies, suggesting that these effects may not be significant (43). In a single existing cross-sectional study involving 1,109 individuals with stroke, a non-linear U-shaped correlation was found between selenium intake and the risk of post-stroke depression (PSD) (20).

Previous animal experiments have shown that reactive oxygen species, produced during stroke, can have detrimental effects on the brain through oxidative stress, which is a key mechanism for inducing PSD (44). Prolonged exposure to high levels of oxidative stress may counteract the protective effects of dietary selenium intake against stroke, which may explain the discrepancy in the non-stroke populations. Previous studies have suggested that antioxidant nutrients obtained from dietary sources enhance the body’s defense mechanisms against oxidation, help eliminate excess free radicals, and reduce oxidative stress (45). Additionally, stroke can disrupt the balance of the intestinal microflora in patients, which can further contribute to brain injury through changes in T cell homeostasis, pro-inflammatory responses, and oxidative stress (46). Furthermore, certain medications commonly used for stroke treatment, such as antiplatelet agents and statins, may exert protective effects by reducing harmful metabolic changes in the ischemic brain. However, it is important to note that the assumption of relatively stable dietary habits over time in this study may be a potential limitation, as dietary habits can vary especially in emotionally negative participants. Further investigations are necessary to better understand the underlying pathophysiology of the relationship between dietary selenium intake, stroke, and its effects on depression. Several mechanisms may explain the association between dietary selenium intake and depression in individuals with and without stroke. A biologically plausible pathway for depression involves inflammation, oxidative stress, and lower antioxidant levels (47). Several hypotheses have been proposed; however, nothing is known about the processes by which dietary selenium affects the development of depression. First, selenium is generally present in the body in the form of selenoproteins, such as glutathione peroxidases and thioredoxin reductases, whose most important role is to provide protection against lipoperoxidation and oxidative cell damage (48, 49). Second, according to current research, the glutathione antioxidant system is known to be associated with the pathophysiology of mood disorders (50). Finally, by controlling the expression of selenoprotein genes and decreasing the production of C-reactive proteins, selenium constraints the activation of NF-kappaB, which can reduce inflammation (51). Our threshold effect in non-stroke individuals (128.4 mcg/d) corresponds to 1.8 × the US RDA (55 mcg/d), suggesting current recommendations may underestimate optimal neuropsychiatric selenium requirements.

### 5.1 Limitations

In this research, the association between selenium intake and depression in stroke and non-stroke patients was explored after adjusting for potential variables such as baseline characteristic. This research provides several strengths. Methodological strengths enhance the validity of these findings: (1) Nationally representative NHANES sampling ensuring generalizability; (2) Comprehensive adjustment for 15 potential confounders including inflammatory biomarkers; (3) Restricted cubic spline modeling revealing non-linear relationships; (4) Sensitivity analyses confirming robustness across multiple model specifications. However, this study has some limitations. First, cross-sectional study could not infer any causal relationships. Second, there is a chance that the questions were misunderstood or that recollection bias existed, because the information gathered during the interviews was self-reported. Third, considering that the dietary data were collected from a self-reported 24-h dietary review, biases in recollection and self-reporting might still exist. If the same subject is resampled in an entirely distinct circle, the findings may not be accurate. In addition, the assumption that dietary habits are relatively stable over time may be biased. The likelihood of this occurrence, however, is extremely low given that participants was chosen using a multistage, stratified probability design and that the NHANES investigates over 5,000 individuals annually from 15 different counties across the nation, which represents a sizable portion of the country’s population. Fourth, key clinical variables such as infarct/hemorrhage size, baseline National Institutes of Health Stroke Scale (NIHSS) scores, and modified Rankin Scale (mRS) at discharge were unavailable in the retrospective dataset. The absence of these variables limits our ability to adjust for baseline stroke severity. The unavailability of discharge mRS scores precludes a direct analysis of early post-stroke recovery patterns and their relationship to long-term outcomes. Future prospective studies should prioritize the systematic collection of these variables to strengthen causal inferences. Despite these limitations, our findings remain robust in characterizing population-level trends and identifying modifiable risk factors (e.g., post-stroke depression) that are actionable in clinical practice. Finally, despite accounting for various confounding variables, we cannot completely exclude the possibility that the connections we observed were caused by unmeasured confounders. Well-designed multicenter randomized controlled trials are required to further validate our findings in light of these limitations.

## 6 Conclusion

In summary, our analyses revealed a nonlinear inverse association between dietary selenium intake and depression prevalence among stroke-free individuals, with optimal risk reduction observed at selenium levels ≥128.4 mcg/d. Notably, stroke status significantly modified the selenium-depression association (Pinteraction = 0.007), suggesting cerebrovascular events may alter selenium’s neuroprotective pathways. Prospective cohort studies with repeated selenium measurements are warranted to establish causal relationships and delineate temporality in populations with and without stroke.

## Data Availability

The raw data supporting the conclusions of this article will be made available by the authors, without undue reservation.
