# Supplementary material for: Association between dietary selenium intake and depression in patients with or without stroke: a cross-sectional study
Source: Front Nutr. 2025 Jun 5;12:1493603. doi: 10.3389/fnut.2025.1493603 (PMC12176551; doi:10.3389/fnut.2025.1493603)
Supplement: Supplementary file 1 [file Table_1.docx]

Supplementary Table S1: Weighted univariate analysis adjusted for covariables, NHANES 2011-2018

| **Variable** | **OR 95CI** | **P-value** |
| --- | --- | --- |
| **Age (years)** | |  |
| 20~40 | 1 |  |
| 40~60 | 1.081 (0.639~1.83) | 0.772 |
| >60 | 0.725 (0.384~1.367) | 0.320 |
| **Gender, n (%)** | |  |
| Male | 1 |  |
| Female | 1.578 (0.976~2.552) | 0.063 |
| **Race, n (%)** | |  |
| Mexican American | 1 |  |
| Other Hispanic | 1.459 (0.45~4.726) | 0.529 |
| Non-Hispanic white | 0.869 (0.364~2.072) | 0.751 |
| Non-Hispanic black | 1.109 (0.382~3.217) | 0.849 |
| Other races | 1.104 (0.357~3.408) | 0.864 |
| **Education level, n (%)** | |  |
| Less than high school | 1 |  |
| High school | 0.659 (0.326~1.332) | 0.246 |
| More than high school | 0.422 (0.226~0.787) | 0.007 |
| **Marital status, n (%)** | |  |
| Married/Living with partner 1 | | |
| Divorced/separated/widowed | 0.76 (0.367~1.572) | 0.456 |
| Never married | 0.451 (0.263~0.771) | 0.004 |
| **Body mass index, kg/m2** | | |
| ＜25 | 1 |  |
| 25~30 | 0.88 (0.454~1.707) | 0.705 |
| ≥30 | 1.604 (0.903~2.848) | 0.107 |
| **Physical activity, n (%)** | |  |
| High (≥600) | 1 |  |
| Low (＜600) | 0.719 (0.409~1.263) | 0.251 |
| **Energy intake, n (%)** |  |  |
| Tertile1 | 1 |  |
| Tertile2 | 0.751 (0.429~1.316) | 0.318 |
| Tertile3 | 0.649 (0.367~1.149) | 0.138 |
| **Smoking status, n (%)** | |  |
| No | 1 |  |
| Yes | 2.389 (1.478~3.861) | <0.001 |
| **Alcohol consumption, n (%)** | | |
| No | 1 |  |
| Yes | 1.391 (0.595~3.256) | 0.446 |
| **Hypertension, n (%)** | |  |
| No | 1 |  |
| Yes | 1.512 (0.947~2.413) | 0.083 |
| **Hyperlipidemia, n (%)** | |  |
| No | 1 |  |
| Yes | 1.331 (0.787~2.249) | 0.286 |
| **Diabetes, n (%)** | |  |
| No | 1 |  |
| Yes | 1.519 (0.784~2.941) | 0.216 |
| **Sleeping disorders, n (%)** | | |
| Yes | 1 |  |
| No | 0.255 (0.152~0.427) | <0.001 |
| **Stroke** |  |  |
| Yes | 1 |  |
| No | 0.361 (0.136~0.953) | 0.04 |
| **Total cholesterol (mg dL^−1^)** | 1.001 (0.996~1.007) | 0.629 |
| **Total triglyceride (mg dL^−1^)** | 1.001 (0.998~1.004) | 0.457 |
| **HDL-C (mg dL^−1^)** | 0.988 (0.973~1.004) | 0.143 |
| **LDL-C (mg dL^−1^)** | 1.002 (0.991~1.012) | 0.767 |

Supplementary Table S2: Associations between dietary selenium intake and depression among US adults, NHANES 2011- 2018.

|  | ≥20 years adults (n=15018) | | |  |  |  |  |
| --- | --- | --- | --- | --- | --- | --- | --- |
|  | β (95% CI) |  |  |  | OR (95% CI) | |  |
| Factors | Crude | Model 1 | Model2 |  | Crude | Model 1 | Model2 |
| Selenium intake (per-SD change) | -0.008 (-0.01~-0.007)** | -0.005 (-0.007~-0.004)** | -0.01 (-0.012~-0.008)** | | 0.993 (0.987~0.998)*** | | 0.991 (0.983~0.999)**** |
| Subgroups | |  |  |  |  |  |  |
| Quartile 1 | Reference | Reference | Reference |  | Reference | Reference | Reference |
| Quartile 2 | -0.717 (-0.909~-0.525)** | -0.586 (-0.775~-0.397)** | -0.695 (-0.884~-0.506)** | | 0.682 (0.38~1.224) | 0.72 (0.396~1.31) | 0.696 (0.365~1.328) |
| Quartile 3 | -1.107 (-1.297~-0.916)** | -0.875 (-1.066~-0.684)** | -1.178 (-1.381~-0.975)** | | 0.439 (0.23~0.84)**** | 0.486 (0.248~0.951)**** | 0.401 (0.185~0.868)**** |
| Quartile 4 | -1.067 (-1.257~-0.877) | -0.749 (-0.948~-0.55)** | -1.268 (-1.508~-1.027)** | | 0.462 (0.244~0.872)**** | 0.53 (0.266~1.056) | 0.395 (0.158~0.984)**** |
| P-trend | <0.001 | <0.001 | <0.001 |  | 0.0063 | 0.0328 | 0.0204 |

*Crude model: Unadjusted crude model.*

*Model 1: Adjusted for age, gender, race, education, BMI, and marital status.*

*Model 2: Adjusted for variables in model 1 plus smoking status, alcohol consumption, diabetes, hypertension, sleeping disorders and energy intake.*

*Weighted by: 24-hour dietary recall*

**P< 0.0001, **P< 0.001, ***P< 0.01, ****P< 0.05*

Supplementary Table S3: Subgroup analyses between dietary selenium intake and risk of depression in patients with or without stroke among US adults, NHANES 2011-2018. Dietary selenium exclude (X±3SD)

| **Variable** | **With stroke (n=545)** | |  | **Without stroke (n=14473)** | |  | **P for interaction** |
| --- | --- | --- | --- | --- | --- | --- | --- |
|  | **OR 95% CI** | **P-value** |  | **OR 95% CI** | **P-value** |  |  |
| Dietary selenium intake (mcg/d) | 0.997 (0.988~1.005) | 0.459 |  | 0.993 (0.991~0.995) | <0.001 |  | 0.059 |
| Subgroups |  |  |  |  |  |  | 0.008 |
| Quartile 1 | Ref. |  |  | Ref. |  |  |  |
| Quartile 2 | 1.64 (0.865~3.112) | 0.13 |  | 0.684 (0.576~0.812) | <0.001 |  |  |
| Quartile 3 | 1.383 (0.642~2.979) | 0.408 |  | 0.53 (0.435~0.645) | <0.001 |  |  |
| Quartile 4 | 0.972 (0.377~2.509) | 0.953 |  | 0.468 (0.368~0.596) | <0.001 |  |  |
| Trend test |  | 0.946 |  |  | <0.001 |  |  |

*Crude model: Unadjusted crude model.*

*Model: Adjusted for age, gender, race, education, BMI, marital status, smoking status, alcohol consumption, diabetes, hypertension, sleeping disorders and energy intake.*

**P< 0.0001, **P< 0.001, ***P< 0.01, ****P< 0.05*

Supplementary Table S4: Subgroup analyses between dietary selenium intake and risk of depression in patients with or without stroke among US adults, NHANES 2011-2018. Dietary selenium exclude (X±2SD)

| **Variable** | **With stroke (n=545)** | |  | **Without stroke (n=14473)** | |  | **P for interaction** |
| --- | --- | --- | --- | --- | --- | --- | --- |
|  | **OR 95% CI** | **P-value** |  | **OR 95% CI** | **P-value** |  |  |
| Dietary selenium intake (mcg/d) | 0.999 (0.99~1.008) | 0.815 |  | 0.991 (0.989~0.994) | <0.001 |  | 0.006 |
| Subgroups |  |  |  |  |  |  | 0.002 |
| Quartile 1 | Ref. |  |  | Ref. |  |  |  |
| Quartile 2 | 1.426 (0.726~2.802) | 0.303 |  | 0.739 (0.621~0.879) | <0.001 |  |  |
| Quartile 3 | 1.762 (0.838~3.706) | 0.135 |  | 0.503 (0.41~0.617) | <0.001 |  |  |
| Quartile 4 | 0.918 (0.348~2.421) | 0.863 |  | 0.502 (0.396~0.638) | <0.001 |  |  |
| Trend test |  | 0.723 |  |  | <0.001 |  |  |

*Crude model: Unadjusted crude model.*

*Model: Adjusted for age, gender, race, education, BMI, marital status, smoking status, alcohol consumption, diabetes, hypertension, sleeping disorders and energy intake.*

**P< 0.0001, **P< 0.001, ***P< 0.01, ****P< 0.05*

Supplementary Table S5: Weighted odds ratios (95% confidence intervals) of depression and dietary selenium intake levels in different models among US adults, NHANES 2011- 2018.

|  | **With stroke (n=545)** | |  |  | **Without stroke (n=14473)** | |  |
| --- | --- | --- | --- | --- | --- | --- | --- |
|  | **Crude** | **Model 1** | **Model2** |  | **Crude** | **Model 1** | **Model2** |
| Selenium intake (per-SD change) | 1.006 (0.985~1.027) | 1.005 (0.981~1.029) | 1.005 (0.98~1.03) | | 0.992 (0.986~0.998)*** | 0.993 (0.987~1.01)**** | 0.99 (0.982~0.999)**** |
| Subgroups | |  |  |  |  |  |  |
| Quartile 1 | Reference | Reference | Reference |  | Reference | Reference | Reference |
| Quartile 2 | 1.618 (0.132~19.888) | 1.564 (0.109~22.469) | 1.674 (0.099~28.232) | | 0.654 (0.358~1.195) | 0.689 (0.372~1.277) | 0.698 (0.369~1.318) |
| Quartile 3 | 1.372 (0.086~21.944) | 1.33 (0.066~26.76) | 1.381 (0.059~32.281) | | 0.419 (0.215~0.819)**** | 0.462 (0.231~0.924) | 0.432 (0.211~0.882)**** |
| Quartile 4 | 2.044 (0.137~30.537) | 1.948 (0.096~39.412) | 1.772 (0.073~43.082) | | 0.435 (0.225~0.84)**** | 0.496 (0.243~1.012) | 0.470 (0.226~0.979)**** |
| P-trend | 0.642 | 0.699 | 0.753 |  | 0.005 | 0.024 | 0.018 |

*Data presented are ORs and 95% Cls.*

*Crude model: Unadjusted crude model.*

*Model 1: Adjusted for age, gender, race, education, BMI, and marital status.*

*Model 2: Adjusted for variables in model 1 plus* *smoking status, alcohol consumption, diabetes, hypertension, hyperlipidemia, sleeping disorders, energy intake and serum selenium.*

*Weighted by: 24-hour dietary recall*

**P< 0.0001, **P< 0.001, ***P< 0.01, ****P< 0.05*
