# Supplementary material for: Association between dietary selenium intake and depression in patients with or without stroke: a cross-sectional study
Source: Front Nutr. 2025 Jun 5;12:1493603. doi: 10.3389/fnut.2025.1493603 (PMC12176551; doi:10.3389/fnut.2025.1493603)
Supplement: Supplementary file 2 [file Image_1.pdf]

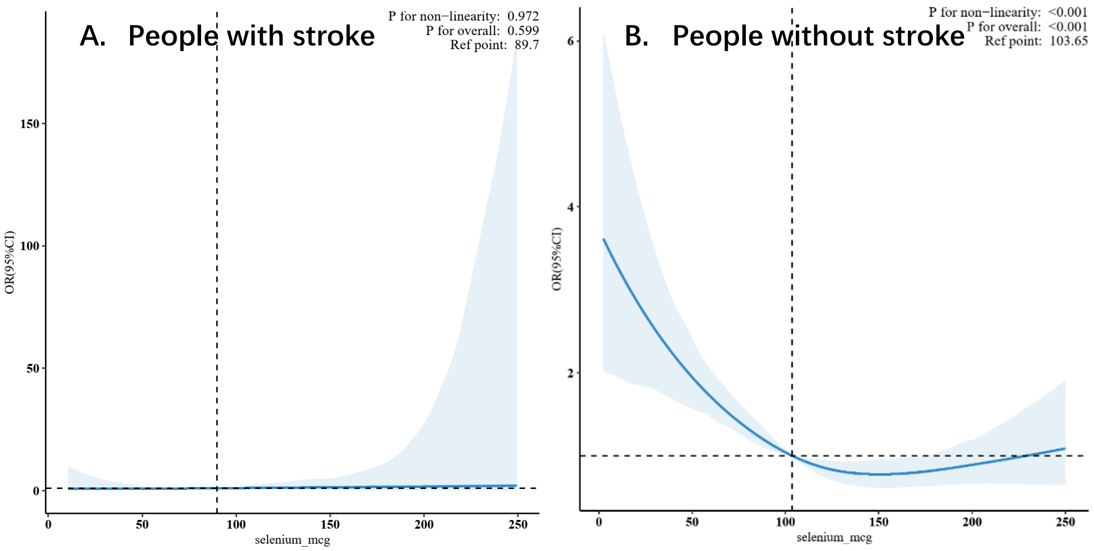

Supplementary Figure 1. Restricted cubic spline model of the weighted odds ratios of dietary selenium intakes with depression with stroke (A) or without stroke (B). Adjusted for age, gender, race, education, BMI, and marital status, smoking status, alcohol consumption, diabetes, hypertension, hyperlipidemia, sleeping disorders, total daily energy intake and serum selenium. The dashed lines represent the 95% confidence intervals.
